# Effects of straw mulch on soil water and winter wheat production in dryland farming

**DOI:** 10.1038/srep10725

**Published:** 2015-06-02

**Authors:** Zhang Peng, Wei Ting, Wang Haixia, Wang Min, Meng Xiangping, Mou Siwei, Zhang Rui, Jia Zhikuan, Han Qingfang

**Affiliations:** 1 Institute of Water-saving Agriculture in Arid Area of China, Northwest A&F University, Yangling, 712100 Shaanxi, China; 2 Key Laboratory of Crop Physi-ecology and Tillage Science in Northwestern Loess Plateau, Minister of Agriculture, Yangling, 712100 Shaanxi, China; 3 Colloge of Agronomy, Northwest A&F University, Yangling, 712100 Shaanxi, China

## Abstract

The soil water supply is the main factor that limits dryland crop production in China. In a three-year field experiment at a dryland farming experimental station, we evaluated the effects of various straw mulch practices on soil water storage, grain yield, and water use efficiency (WUE) of winter wheat (*Triticum aestivum*). Field experiments were conducted with six different mulch combinations (two different mulch durations and three different mulch amounts): high (SM1; 9000 kg ha^−1^), medium (SM2; 6000 kg ha^−1^), and low (SM3; 3000 kg ha^−1^) straw mulch treatments for the whole period; and high (SM4), medium (SM5) and low (SM6) straw mulch treatments during the growth period only, where the control was the whole period without mulch (CK). Throughout the whole growth period of the three-year experiment, the average soil water content in the 0–200 cm soil layer increased by 0.7–22.5% compared with CK, while the WUE increased significantly by 30.6%, 32.7% and 24.2% with SM1, SM2, and SM3, respectively (P < 0.05). The yield increased by 13.3–23.0% when mulch was provided during the growth period, while the WUE increased by 15.2%, 17.2% and 18.0% with SM4, SM5, and SM6, respectively, compared with CK.

Water storage and large variations in inter-annual and intra-annual rainfall are the main constraints that affect agricultural production in the dryland farming areas of northern China where the annual precipitation is 550 mm and evaporation is 1840 mm^1^. The rainfall pattern is highly variable with > 60% of the annual rainfall occurring in the summer months (July to September) and the soil water storage efficiency is less than 30%. The precipitation during the growth period cannot meet with the requirements of winter wheat^2^,^3^. Therefore, the ability to store this rainfall in the soil for later use by crops is an important feature of agriculture in this region^4^, and thus improving the soil water capacity and water use efficiency (WUE) also has significant effects in promoting crop productivity in this region.

The rates of crop straw use for fuel and forage have declined significantly since the 1980s and crop straw is increasingly burned after the harvest, thereby leading to high losses of soil organic substances^5^,^6^, reduced water stability in the entire soil^7^, and increased CO_2_ emissions, which affect the environment^8^. Straw mulch can protect surface farmland soil from the direct impact of rainfall, disconnect the evaporation surface from the capillarity of the subsoil, and reduce the turbulent exchange between the soil air and the atmosphere to effectively inhibit the evaporation of soil water^9^,^10^,^11^. Straw mulch has different effects on soil moisture and crop growth in regions with different climates, soil types, and other conditions^12^. In dryland farming conditions, straw mulch can reduce the latent heat flux in the soil and decrease the rate of evaporation between winter wheat plants, which allows more soil water to be accumulated^13^,^14^,^15^. Duley *et al.*^16^ conducted the first experiments to test the effects of different straw mulch amounts, which demonstrated that the soil water content increased twofold in land covered with straw mulch at a rate of 4500 kg ha^−1^ compared with an untreated control. Hares^17^ showed that the inhibitory effect of straw mulch on soil evaporation with winter wheat during the summer fallow period could reach 63.2%, while Zhao *et al.*^18^ found that the inhibitory rate of straw mulch during the wheat growth period was 21.5% during the wintering to jointing growth stage. Many studies have found that inappropriate mulching amounts and methods can have negative effects, which result in yield reductions^19^,^20^. Unger^21^ and Wicks *et al.*^22^ suggested that different amounts of straw mulch can have various effects on the yield, and that the crop yield is only increased with mulch amounts within certain ranges. Cook *et al.*^23^ also reported that a wheat straw mulch at a rate of 2000–4000 kg ha^−1^ increased the maize yield, whereas a straw mulch of 6000–8000 kg ha^−1^ greatly decreased maize production.

As a typical dryland farming region located in north-central Shaanxi Province, Weibei Highland is known as “the second granary of Shaanxi” because it has a crucial status in ensuring the food supply safety of Shaanxi and China. However, low precipitation, high evapotranspiration, water deficiency, and limited water use are the main factors that lead to low and variable crop yields in this areas^24^,^25^. In the present study, in order to reduce soil water evaporation and increase the WUE, we tested six different straw mulch treatments (two different mulch durations and three different mulch amounts) continuously for three years: high (SM1; 9000 kg ha^–1^), medium (SM3; 6000 kg ha^–1^), and low (SM2; 3000 kg ha^–1^) straw mulch treatments for the whole period; and high (SM4), medium (SM5), and low (SM6) straw mulch treatments during the growth period only, where the control was the whole period without mulch (CK). We investigated the effects of different straw mulch treatments on water conservation, WUE, and the wheat yield to provide basic information that may facilitate the appropriate selection of mulch methods in these regions.

## Results

### Effects of straw mulch on soil water storage in different growth stages

**Figure 1:**
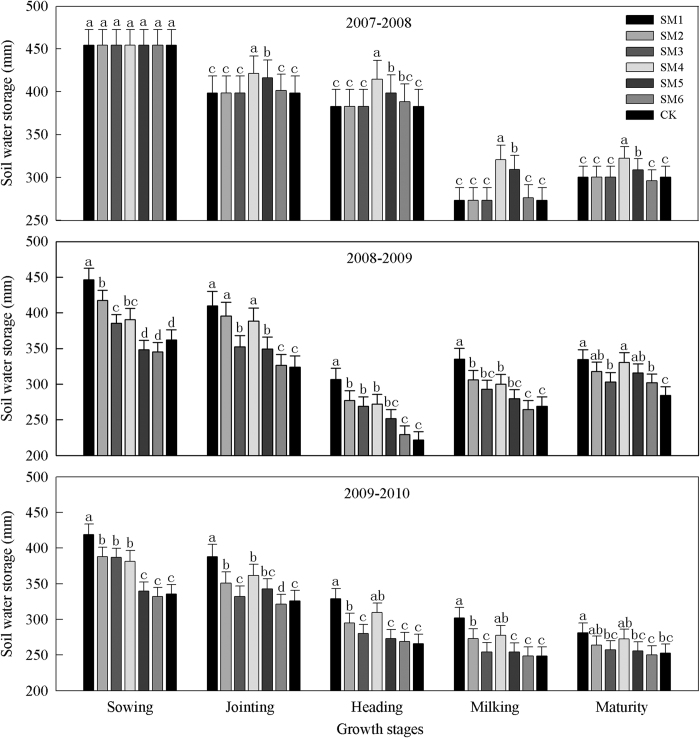
Soil water storage in 0-200 cm soil layer under different straw mulch treatments (mm) during 2007-2010. Note: CK, no straw mulch; SM1, straw mulch at a high rate of 9000 kg ha^−1^ in whole-period of winter wheat; SM2, straw mulch at a middle rate of 6000 kg ha^−1^ in whole-period of winter wheat; SM3, straw mulch at a low rate of 3000 kg ha^−1^ in whole-period of winter wheat; SM4, straw mulch at a high rate of 9000 kg ha^−1^ in growth-period of winter wheat; SM5, straw mulch at a middle rate of 6000 kg ha^−1^ in growth -period of winter wheat; SM6, straw mulch at a low rate of 3000 kg ha^−1^ in growth -period of winter wheat. Bars with different lower case letters indicate significant differences at *P* < 0.05. Error bars are the standard deviation.

The straw mulch treatments increased soil water storage compared with CK in nearly all of the winter wheat growth stages and water storage increased with the mulch amounts in the same mulch period (Fig. 1).

#### Sowing stage

Differences in precipitation during the summer fallow period (335.2 mm in 2008, 243.1 mm in 2009, and 195.8 mm in 2010) meant that soil water storage at the sowing stage was significantly (P < 0.05) higher under the whole-period straw mulch treatments (SM1, SM2, and SM3) compared with CK at the 0–200 cm depth, i.e., by 23.6–84.5 mm in 2008, and by 51.0–83.2 mm in 2009, whereas under the growth-period mulch treatments, only SM4 increased significantly (P < 0.05), i.e., by 28.6 in 2008 and 45.4 mm in 2009. With the same mulch amounts, the soil water storage with the whole-period straw mulch treatments was higher than that with the growth-period mulch treatments, but the difference decreased as the rainfall declined, i.e., 55.1 mm in 2008, and 47.0 mm in 2009.

#### Jointing, Heading, and Milking stages

In the middle growth stages (jointing, heading, and milking stages), the mean soil water storage rates in the 0–200 cm depth with the growth-period mulch treatments, i.e.,SM4, SM5, and SM6, were 33.6, 20.1, and 1.4 mm higher than CK during 2007–2008, as well as 48.6, 22.1, and 1.8 mm higher during 2008–2009, and 35.9, 9.7, and 0.8 mm during 2009–2010, respectively. However, there were no significant differences between SM6 and CK in all the middle growth stages. The soil water storage with the three mulch treatments was ranked in the order: SM4 > SM5 > SM6. The soil water storage rates in the 0–200 cm depth with the whole-period straw mulch treatments (SM1, SM2, and SM3) were similar to those with the growth-period mulch treatments, where the three mulch treatments was ranked in the order: SM1 > SM2 > SM3 during all the middle growth stages, SM1, SM2, and SM3 were 79.0, 54.8 and 33.2 mm higher than CK during 2008–2009, and 59.4, 26.0 and 8.5 mm during 2009–2010, respectively. Using the same mulch amounts, the two-year average soil water storage with the whole-period straw mulch treatments was higher than that with the growth-period mulch treatments in each of the middle growth stages, i.e., SM1 was 27.0 mm higher than SM4, SM2 was 24.5 mm higher than SM5, and SM3 was 20.3 mm higher than SM6.

#### Maturity stage (harvest stage)

In the later growth stage (maturity stage), the rainfall was 105.7 mm in 2008, when the water storage only increased significantly (P < 0.05) with the SM4 and SM5 treatments, i.e., by 21.8 and 8.5 mm, respectively, compared with CK. In 2009, when the rainfall (46.8 mm) was <50% of that in 2008, the mean soil water storage with the straw mulch treatments increased significantly by 32.8 mm, the water storage increased gradually with greater amounts of straw mulch, and the soil water storage with the whole-period straw mulch treatments was higher than that with the growth-period mulch treatments, i.e., SM1 was 4.2 mm higher than SM4, SM2 was 2.2 mm higher than SM5, and SM3 was 0.7 mm higher than SM6. In 2010, when the annual rainfall was very low (442.6 mm), the soil water storage with all treatments was lower than that in 2008–2009 (Fig. 1) during this stage. The soil water storage was slightly higher with straw mulch treatments compared with CK at the 0–200 cm depth, i.e., a significant increase of 28.4 mm using SM1 treatments compared with CK, but no significant differences in soil water storage between SM2, SM3, SM4, SM5, SM6, and CK.

### Effect of straw mulch on soil water storage in different soil layers

#### Soil moisture in the 0-20 cm soil layer

The effect of soil moisture in the 0–20 cm soil layer is critical for the shooting of winter wheat in arid areas. Table 1 shows the soil water content dynamics in the 0–20 cm soil layers with all treatments during the five winter wheat growing stages in 2007–10 (in the first year of the study, 2007–08, we only tested three growth-period treatments, i.e., SM4, SM5, and SM6, and we only measured four growing stages). During 2007-2008, the fallow period rainfall was 335.2 mm (Table 2), which resulted in a high soil water content during the sowing stage (20.7%), but it decreased slightly with the growth process. During 2008-2009, the fallow period rainfall (243.1 mm) was lower than that in 2007-2008. The mean soil water content with all straw mulch treatments was 13.6% higher than CK treatment in the sowing stage. The wheat entered the jointing stage in early April and the heading stage in late April, and in these stages, the rainfall was 84.4 and 12.8 mm, respectively. Compared with CK, the average soil water content during the jointing and heading stages were 30.2% and 22.1% higher with the whole-period straw mulch treatments (SM1, SM2, and SM3), and 26.4% and 17.1% higher with the growth-period straw mulch treatments (SM4, SM5, and SM6), respectively. The wheat was in the milking stage from mid-May to mid-June, where the rainfall during this stage was 136.5 mm, but the rainfall increased and the soil water content recovered with each treatment (Table 1), and thus the differences among treatments were not significant. Wheat entered the maturity stage in mid-June when the mean soil water contents were 21.7% and 17.0% greater with the whole-period straw mulch and growth-period straw mulch treatments, respectively, compared with CK. With the same mulch amounts, the average soil water storage with whole-period straw mulch treatments was higher than that under growth-period mulch treatments in all growth stages, i.e., SM1 was 7.6% higher than SM4, SM2 was 10.4% higher than SM5, and SM3 was 8.2% higher than SM6. During 2009-2010, the fallow period rainfall was only 195.8 mm and the straw mulch treatments increased the soil water content significantly (P < 0.05) in the 0–20 cm depth compared with CK (by 15.9%). In the middle grow stages (jointing, heading, and milking), wheat growth entered the vigorous growth period and the crop water consumption increased strongly. During this stage, the rainfall was low (190.1 mm), and the soil water content dropped to its minimum in the milking stages (Table 1), where the mean soil water contents were 24.1% and 16.2% greater with the whole-period straw mulch and growth-period straw mulch treatments, respectively, compared with CK. In the maturity stage, with 56.7 mm rainfall, the soil water content recovered with all treatments and the mean soil water contents with the straw mulch treatments was 8.4% higher than that with CK. Using the same amounts of mulch, the average soil water storage with the whole-period straw mulch treatments was 4.3% higher than that with the growth-period mulch treatments.

#### Soil water storage in the 0-100 cm soil layer

**Figure 2:**
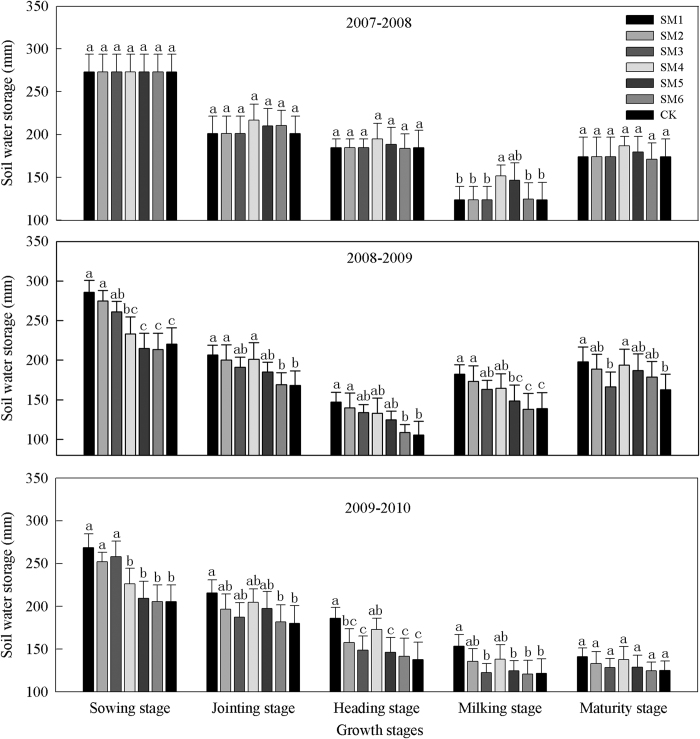
Soil water storage in 0-100 cm soil layer under different straw mulch treatments (mm) during 2007-2010. Note: CK, no straw mulch; SM1, straw mulch at a high rate of 9000 kg ha^−1^ in whole-period of winter wheat; SM2, straw mulch at a middle rate of 6000 kg ha^−1^ in whole-period of winter wheat; SM3, straw mulch at a low rate of 3000 kg ha^−1^ in whole-period of winter wheat; SM4, straw mulch at a high rate of 9000 kg ha^−1^ in growth-period of winter wheat; SM5, straw mulch at a middle rate of 6000 kg ha^−1^ in growth -period of winter wheat; SM6, straw mulch at a low rate of 3000 kg ha^−1^ in growth -period of winter wheat. Bars with different lower case letters indicate significant differences at *P* < 0.05. Error bars are the standard deviation.

During 2007-2008, compared with CK, the straw mulch treatments did not increase the soil water storage significantly in the 0–100 cm soil layer during all growth stages (Fig. 2), expect the milking stage (SM4 significantly increased by 28.0 mm), which may have been related to the higher rainfall during this period (335.2 mm in the fallow period and 105.7 mm in the maturity stage). During 2008-2009, the soil water storage was closely related to the rainfall in each growth stage (Fig. 2). In the sowing stage, the soil water storage (0–100 cm) increased significantly (P < 0.05) with the SM1, SM2, and SM3 treatments by 65.6, 54.3, and 40.6 mm, respectively, compared with CK, but there were no significant differences between SM4, SM5, SM6, and CK. In the middle stages (jointing, heading, and milking), compared with CK, the average soil water storage rates with the whole-period and growth-period straw mulch treatments increased by 31.1 and 17.1 mm at the jointing stage, respectively, by 34.8 and 16.5 mm at the heading stage, and by 34.0 and 11.7 mm at the milking stage. In the maturity stage, only SM1 and SM4 increased the soil water storage significantly (P < 0.05), i.e., by 34.9 and 31 mm, respectively, compared with CK. During 2009-2010, the rainfall amounts in each growth stage were lower than those in 2007-2008 and 2008-2009. The mean soil water storage with the whole growth-period mulch treatments was higher than that with CK, i.e., 63.1, 46.9 and 52.7 mm higher with SM1, SM2, and SM3 at the sowing stage, respectively, as well as 38.6, 16.9, and 6.4 mm higher at the middle stage (jointing, heading, and milking), and 16.2, 8.2 and 3.6 mm higher at the maturity stage. And the mean soil water storage were 22.0, 7.3 and 1.0 mm greater with SM4, SM5, and SM6, respectively, compared with CK treatment during the whole wheat growth-period, but the differences between the growth-period mulch treatments were not significant. Using the same amounts of mulch, the average soil water storage with the whole-period straw mulch treatments was 15.0 mm higher than that with the growth-period mulch treatments.

#### Soil water storage in 100-200 cm soil layer

**Figure 3:**
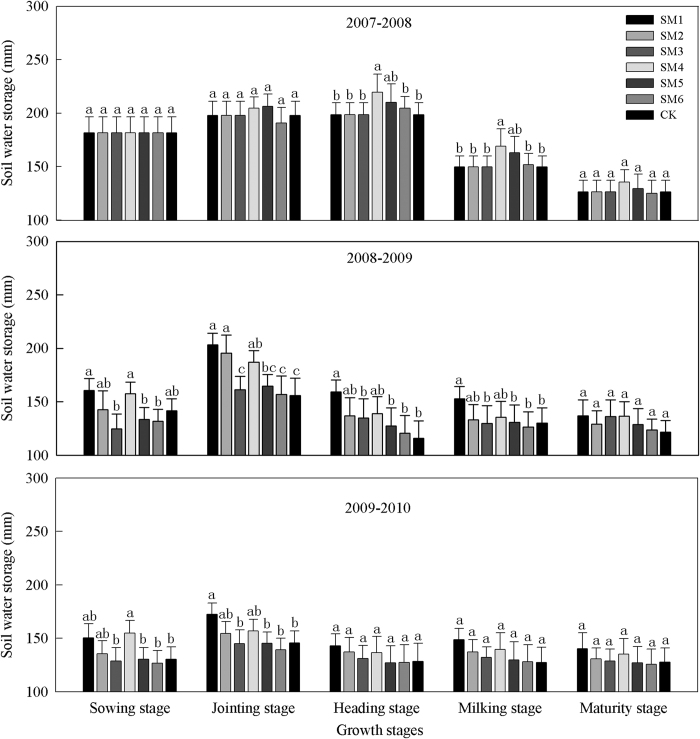
Soil water storage in 100-200 cm soil layer under different straw mulch treatments (mm) during 2007-2010. Note: CK, no straw mulch; SM1, straw mulch at a high rate of 9000 kg ha^−1^ in whole-period of winter wheat; SM2, straw mulch at a middle rate of 6000 kg ha^−1^ in whole-period of winter wheat; SM3, straw mulch at a low rate of 3000 kg ha^−1^ in whole-period of winter wheat; SM4, straw mulch at a high rate of 9000 kg ha^−1^ in growth-period of winter wheat; SM5, straw mulch at a middle rate of 6000 kg ha^−1^ in growth -period of winter wheat; SM6, straw mulch at a low rate of 3000 kg ha^−1^ in growth -period of winter wheat. Bars with different lower case letters indicate significant differences at *P* < 0.05. Error bars are the standard deviation.

In the 100–200 cm soil layer, the soil water storage was stable in the different winter wheat growth stages (Fig. 3). During 2007-2008, compared with CK, the mean soil water storage with all mulch treatments were only increased by 11.4, 7.3 and 0.1 mm during the all wheat growth stages During 2008-2009, the highest soil water storage was at the jointing stage with all of the mulch treatments had, where the water storage with SM1, SM2 and SM4 increased by 47.5, 39.7, and 31.1 mm, respectively. The changes in water storage during 2009-2010 were consistent with those in 2008-2009.

### Effect of straw mulch on WUE and winter wheat production

The wheat yield differed with the variation in precipitation among the three experimental seasons (Table 3). During 2007-2008, the annual rainfall was 603.6 mm and water was not the main limiting factor for the wheat yield. However, compared with CK, SM4 and SM5 decreased the wheat yield by 5.9% and 6.6%, respectively, whereas SM6 increased the yield by 5.9%, which may occurred because the high straw mulch amounts in a rainy year lowered the topsoil temperature. During 2008-2009 and 2009-2010, the rainfall rates were 523.6 and 442.6 mm, respectively, and the wheat yields with all treatments were lower than those in 2007-2008. Compared with CK, the whole-period mulch treatments (SM1, SM2, and SM3) significantly (P < 0.05) increased the wheat yields by 41.1-65.7% in 2009 and by 25.8-32.6% in 2010, while the growth-period mulch treatments (SM4, SM5, and SM6) significantly increased the yields by 30.1–30.7% in 2009, and there were no significantly increases of 4.0–17.7% in 2010. The two-year average wheat yields increased with all treatments when using the whole-period mulch, i.e., 17.4% higher than that with the growth-period mulch.

The water consumption was closely related to the rainfall in different years (Table 4). During 2007-2008, the water consumption rates with SM4 and SM5 were 21.8 and 8.5 mm lower than that with CK, respectively, whereas the rate increased by 4.2 mm with SM6 compared with CK. During 2008-2009, the rainfall in the wheat growth period was 294.1 mm and the water consumption was significantly higher with the whole-period mulch treatments than CK, i.e., 34.2, 22.1 and 5.2 mm higher with SM1, SM2, and SM3, respectively. The water consumption rates with the growth-period mulch treatments were lower than that with CK, i.e., 17.4, 44.8, and 34.4 mm lower with SM4, SM5, and SM6, respectively. During 2009-2010, when the growth period rainfall decreased the water consumption with all treatments was lower than that in 2008-2009. Compared with CK, the water consumption rates with SM1, SM2, SM3, SM4, and SM5 were increased by 54.7, 40.9, 46.5, 25.5, and 0.6 mm, respectively. The mean water consumption was increased by all treatments with whole-period mulch, i.e., 39.2 mm higher than that with the growth-period mulch treatments.

During 2007-2008, the wheat yield was the highest among the three experimental years, and the WUE was highest with all treatments, but there were no significant differences between the straw mulch treatments and CK (Table 4). During 2008-2010, the rainfall was lower than that in 2007-2008, and the WUE with all treatments decreased by 40.1-46.9%. Compared with CK, the whole-period mulch treatments (SM1, SM2, and SM3) significantly increased the WUE by 39.2–51.6% in 2009 and by 8.5–16.7% in 2010, while the growth-period mulch treatments (SM4, SM5, and SM6) significantly increased the WUE by 36.6–48.2% in 2009 and by 4.5–8.5% in 2010. The two-year average WUE increase with all treatments was 3.2% higher using whole-period mulch compared with growth-period mulch.

## Discussion

Previous studies suggest that the degree of rainfall infiltration and soil water evaporation differs according to the amounts of straw mulch used in mulching treatments and that the soil water-holding capacity differs with various mulching treatments^11^,^26^. Throughout our three-year study, we found that irrespective of the fallow period rainfall rate, the water storage status improved to varying degrees with the straw mulch treatments compared with CK. This improvement probably occurred because crop straw can disconnect the evaporation surface from the capillarity of the subsoil, thereby greatly inhibiting soil water evaporation, which significantly improves the soil water condition^1^,^27^. However, the water storage level was not consistent in both years with all of the mulch treatments, i.e., the levels were lower in 2008-2010 compared with those in 2007-2008 when the amount of rainfall was reduced. Straw mulch is known to be an effective practice that promotes water conservation by reducing soil water evaporation during the summer fallow period^17^, which increases crop yields^27^. Deng *et al.*^28^ reported that in a dryland farmland area in Northern China, the precipitation during the wheat growth period only accounted for 65%–95% of the actual water consumption and 5%–35% of the consumed water was obtained from the soil water stored before sowing. In our study, we showed that the whole-period mulch treatments facilitated more effective storage of summer rainfall in the soil, thereby increasing the soil water storage and rainfall storage efficiency compared with the growth-period mulch treatments. This is because straw mulching in the wheat fallow period can increase rainfall infiltration and alleviate soil water evaporation^1^,^17^. Huang *et al.*^1^ and Liu *et al.*^15^ also suggested that the adoption of mulch practices could increase the soil water content and reduce drought problems during the wheat growing season, which is consistent with our findings.

Many studies have shown that the water consumed by winter wheat in different growth stages comes from different soil layers^2^,^29^. Gong *et al.*^29^ demonstrated that straw mulch treatments could significantly increased the soil moisture, where it increased with the amount of mulch. Wang *et al.*^2^ found that the variations in soil moisture differed between the 0–100 cm and 100–200 cm layers and showed that the soil water active layer with straw mulch treatments was the 10–20 cm layer. These results are consistent with our findings. After this three-year study, we found that the whole-period mulch treatments significantly increased the soil water content in the 0–20 cm soil layer compared with CK, thereby indicating that the whole-period mulch treatments could effectively improve the topsoil water condition, which can be provided a suitable water conditions for the emergence of wheat at the seedling stage in drought years. Deng *et al.*^30^ found that in regions with 450–600 mm rainfall, the precipitation provided 75% of the water consumed by wheat from the green to jointing stage, while the remaining water was provided by the stored soil water, i.e., 82.6% from the 0–100 cm soil layer. During the heading to milking stages, precipitation only provided 14% of the water consumed by wheat, and over 80% of the water provide by the soil water stored before the sowing stage in the 100–200 cm layers. In our study, the soil water stored in the 0–100 cm layers was significantly higher with the whole-period mulch treatments compared with CK (without straw mulch), and it was highest at the sowing stage. The water consumption by wheat increased throughout the growth process, but the soil water storage levels in the 100–200 cm layers with the high and medium straw mulch treatments were also higher than those with CK. This soil water could relieve drought stress during the later growth stages of wheat, possibly enhancing the photosynthetic characteristics of wheat and the grain yield^22^,^31^. The whole-period mulch treatments facilitated the more effective storage of summer rainfall in the soil, where the soil water storage levels in the 0–100 cm and 100–200 cm layers were higher than those with the growth-period mulch treatments in each of the wheat growth stages. This was because the growth-period mulch treatments omitted mulching during the fallow period (>60% of the annual rainfall occurs in this period) and the soil water evaporation rate was greater due to high temperatures^31^.

China has a large dryland farming region in the northwest^32^, which is constrained by water deficiency^1^,^28^, and the productivity of grain crops in this regions is affected significantly by water availability and the soil quality^15^,^33^, thus, more effective management of the soil water content could facilitate sustainable production and improve crop yields. Conventional tillage crop production with the removal or burning of residues can lead to adverse conditions for crop growth and yield reductions as a consequence^34^. Straw mulching is regarded as one of the best ways of improving water retention in the soil and reducing soil evaporation^35^,^38^. Zhang *et al.*^36^ reported positive effects on the crop yield and WUE after crop straw mulching, and the wheat yield and WUE increased gradually with the amount of mulch. The yield and water conservation effects were best when the mulch amount was 7500 kg ha^−1^. However, in our study, the effects of straw mulching on the crop yield and WUE were more variable, which may be attributed to differences in the rainfall conditions. The yield and WUE decreased when the rainfall was lower in the three experimental seasons. In the rainy year (2007-2008), the differences in the yield and WUE were not significant between the straw mulch and CK treatments. These results agree with those obtained in recent studies in temperate climates^19^. It is likely that straw mulching makes the soil temperature suboptimal during the early growth stages of wheat^20^. In the normal rainfall year (2008-2010), compared with CK, the straw mulch treatments, especially the medium and high mulch amounts, increased wheat yield and WUE significantly (P < 0.05), which can be attributed mostly to the increase in soil water due to straw mulching in arid and semiarid conditions^1^. Similar effects were obtained by Zhang *et al.*^36^ based on field experiments in China. By contrast, Chen *et al.*^37^ found that when the straw mulching amount reached 6000 kg ha^−1^, it had negative effects on the wheat yield and the increase in the WUE. These differences may be related to variations in the mulch materials, years, and weather conditions.

The effects of field water management practices on water storage are much lower than those caused by variations in precipitation, but small effects in water conservation during the crop’s growing season can greatly affect the wheat yield and evapotranspiration, as well as the WUE. Compared with the growth-period mulch treatments, the whole-period mulch treatments improved both the soil water storage and soil water consumption, thereby increasing the wheat yield significantly (P < 0.05) (Table 3). This was probably because straw mulching during the fallow period reduced soil evaporation, augmented the infiltration of rainwater into the soil^13^,^14^, and enhanced soil water retention^1^. Our study also demonstrated that the WUE was higher when using whole-period mulch treatments compared with growth-period mulch treatments, but the difference was not significant. It is likely that straw mulching during the whole wheat period provided favorable soil moisture conditions for wheat growth, and thus the water consumption with the whole-period mulch treatments was significantly (P < 0.05) higher than that with the growth-period mulch treatments. Similar effects were reported by Cai *et al.*^38^ based on a four-year field study in China.

## Conclusion

In the dryland farming area of northwest China, we found that the effects of straw mulching differed with the variation in precipitation among the three experimental seasons. In a rainy year, there were no significant (P > 0.05) effects of straw mulch on the soil water storage, wheat yield, and WUE. However, in a normal year, straw mulching significantly (P < 0.05) improved the soil water conditions, increased the wheat yield, and increased the WUE. In addition, compared with the growth-period mulch treatments, the whole-period mulch treatments obtained greater improvements, particularly the SM1 and SM2 treatments. Because of constraints on the amount of wheat straw available, we conclude that SM2 (6000 kg ha^−1^ straw mulching during the whole period) is the most effective treatment for improving wheat production in the dryland farming area of northwest China.

## Materials and Methods

### Experimental Site

A 4-year field experiment was conducted with winter wheat between 2007-2010 at the Dryland Farming Experimental Station of Northwest A&F University, located in Ganjing Town, Heyang County, Shaanxi Province (35°15′N, 110°18′E, 910 m altitude) in northwestern China. The mean annual temperature was 10.5 □. The total annual sunshine was 2528 h and the frost-free period was 169 ~ 180 days. The long-term mean annual rainfall at the site was 550 mm, and the mean anual evaporation was 1832.8 mm. Most of the rainfall occurred from July to September.

The experimental field was flat according to the FAO/UNESCO Soil Classification^39^, and the soil was a Chernozem (dark loessial soil) with 26.8% sand, 41.9% silt, and 21.3% clay. An analysis of soil samples (0-60 cm depth) taken from the same experimental area in September 2007 were showed in Table 5.

### Experimental design and methods

The experiment was a two-factor randomized block with three replicates. Each plot was 3 m wide and 4 m long, and the same plots were used for the three years and the treatments were the same on each plot. The experiment included two mulch periods (growth-period and whole-period mulch) and three straw mulch amounts (high: 9000 kg ha^−1^, medium: 6000 kg ha^−1^, and low: 3000 kg ha^−1^), seven treatments were initiated in 2007: SM1, SM2 and SM3 represented high, medium, and low straw mulch amount treatments in whole-period mulch, and SM4, SM5 and SM6 represented high, medium, and low straw mulch amount treatments in growth-period mulch, and the whole-period without mulch served as control (CK).

For whole-period mulch treatments, the wheat straw was mulched manually in the field after the harvests in 2008-2010, removed for sowing in September, and recovered after sowing done; for growth-period mulch treatments, the straw was mulched manually after sowing done in September, straw removed and the field was fallow with no tillage after the harvests in 2008–2010 until the sowing in September in next year.

No-tillage was applied in the fallow period, where a 5–8 cm wheat stubble height was left after the winter wheat was harvested, and the wheat was directly drilled in all plots in late September.

Winter wheat (cv. Jinmai 47) was sown at a rate of 150 kg ha^−1^, on 18 September 2007, 19 September 2008, and 17 September 2009, using an Amozone NT 250 drill with chisel-type openers and depth-controlling press wheels at a row spacing of 20 cm. Ten days before sowing, a basis fertilizer containing 150 kg N ha^−1^, 120 kg P ha^−1^, and 90 kg K ha^−1^ was spread evenly over each plot and plowed into the 15–20 cm soil layer. For each crop cycle, no artificial irrigation was provided during the years of the experiment, and manual weeding was undertaken as required during the experiment period. Wheat was harvested on 17 June 2008, 21 June 2009, and 18 July 2010.

### Measurements, calculations and statistical data analysis

The rainfall data were recorded using a standard weather station located at the experimental site (Table 2).

Between 2007 and 2010, the soil water content was determined in each plot in each growth stages of winter wheat by taking three random soil core samples using a 54 mm diameter steel core-sampling tube, which was driven manually to a depth of 2.0 m during each growing season (from October to June of next year) and fallow periods (June to September in the next year). The soil cores were weighed wet, dried in a fan-assisted oven at 105 °C for 48 h, and weighed again to determine the soil water content 40. The gravimetric water content was multiplied by soil bulk density to obtain the volumetric water content. The soil water storage was calculated for a 2.0-m profile by multiplying the mean soil volumetric water content by the soil profile depth.

The grain yield was determined at a water content of 12% after manually harvesting the three central rows with a length of 2-m taken randomly from each plot. The water use efficiency was estimated as the grain yield divided by the growing season evapotranspiration (E)^41^, as follows:


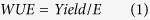


where E was calculated as^42^:


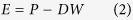


where P is the growing season rainfall and DW is the change in the stored soil water for the soil profile (0–2.0 m depth) between planting and harvest.

The mean values were calculated for each measurement and ANOVA was used to compare the effects of different treatments on the measured variables. If the F–value was significant (*P* < 0.05), multiple comparisons of annual mean values were performed based on the least significant difference (LSD). SPSS 13.0 was used for all statistical analyses.

## Additional Information

**How to cite this article**: Peng, Z. *et al.* Effects of straw mulch on soil water and winter wheat production in dryland farming. *Sci. Rep.* **5**, 10725; doi: 10.1038/srep10725 (2015).

## Figures and Tables

**Table 1:** Soil moistures of 0-20 cm soil layer with different straw mulch treatments in the main growth period of winter wheat (%).

**Year**	**Growth**	**Treatments**						
	**stages**	**SM1**	**SM2**	**SM3**	**SM4**	**SM5**	**SM6**	**CK**
2007	Sowing	—	—	—	20.7	20.7	20.7	20.7
-	Heading	—	—	—	15.6 ± 0.3a	15.2 ± 0.2a	15.1 ± 0.7a	15.1 ± 0.8a
2008	Milking	—	—	—	9.6 ± 0.4a	9.6 ± 0.5a	9.2 ± 0.3a	9.2 ± 0.4a
	Maturity	—	—	—	19.8 ± 0.3a	19.3 ± 1.0a	19.1 ± 0.3a	19.0 ± 1.5a
2008	Sowing	20.6 ± 0.6^b^a^a^	20.5 ± 1.0a	20.0 ± 0.2ab	19.0 ± 0.6bc	18.5 ± 0.8c	18.0 ± 0.9cd	17.1 ± 0.5d
-	Jointing	15.5 ± 0.8a	14.6 ± 0.9ab	13.0 ± 0.3cd	13.9 ± 0.9bc	12.2 ± 0.4d	11.6 ± 0.8de	10.2 ± 1.3e
2009	Heading	9.9 ± 0.5a	9.2 ± 0.4ab	7.8 ± 1.1bc	7.7 ± 1.4c	7.1 ± 0.7cd	6.9 ± 0.5cd	6.0 ± 0.8d
	Milking	17.5 ± 0.6a	17.3 ± 0.7a	17.0 ± 0.9a	17.3 ± 0.3a	17.2 ± 1.0a	16.7 ± 1.2a	16.3 ± 0.3a
	Maturity	16.9 ± 0.3a	16.7 ± 0.3ab	16.4 ± 0.6ab	16.8 ± 0.2ab	15.9 ± 0.8ab	15.4 ± 1.1b	13.7 ± 1.4c
2009	Sowing	20.7 ± 0.2a	20.4 ± 0.2a	20.1 ± 0.7a	19.2 ± 0.3b	18.6 ± 0.3bc	18.5 ± 0.4c	16.9 ± 0.7d
-	Jointing	15.3 ± 0.2a	13.6 ± 0.5b	12.9 ± 0.7bc	14.0 ± 0.9ab	13.5 ± 0.2b	11.7 ± 1.3cd	10.7 ± 1.3d
2010	Heading	12.1 ± 0.8a	10.6 ± 0.3ab	9.9 ± 1.2bc	11.8 ± 1.1a	9.4 ± 0.3bc	9.0 ± 0.7c	8.6 ± 0.7c
	Milking	8.9 ± 0.7a	8.2 ± 0.1abc	7.9 ± 0.8bc	8.4 ± 0.4ab	7.8 ± 0.4bc	7.5 ± 0.4bc	7.4 ± 0.6c
	Maturity	11.8 ± 0.6a	11.5 ± 1.3ab	10.9 ± 0.5abc	11.3 ± 0.1abc	11.0 ± 0.4abc	10.5 ± 0.0bc	10.3 ± 0.1c

Note: CK, no straw mulch; SM1, straw mulch at a high rate of 9000 kg ha^−1^ in whole-period of winter wheat; SM2, straw mulch at a middle rate of 6000 kg ha^−1^ in whole-period of winter wheat; SM3, straw mulch at a low rate of 3000 kg ha^−1^ in whole-period of winter wheat; SM4, straw mulch at a high rate of 9000 kg ha^−1^ in growth-period of winter wheat; SM5, straw mulch at a middle rate of 6000 kg ha^−1^ in growth -period of winter wheat; SM6, straw mulch at a low rate of 3000 kg ha^−1^ in growth -period of winter wheat.

^a^Different lower case letters in the same line indicate significant differences at *P* < 0.05.

^b^Mean ± standard deviation.

**Table 2:** Distribution of monthly precipitation (mm) at the experimental site during the years 2007–2010.

**Year**	**Fallow period**			**Wheat growing season**									**Annual**
	**July**	**Aug.**	**Sep.**	**Oct.**	**Nov.**	**Dec.**	**Jan.**	**Feb.**	**Mar.**	**Apr.**	**May**	**June**	
2007-2008	196.5	83.2	55.5	48.3	1.6	7	29.1	8.3	13	31.9	23.5	105.7	603.6
2008-2009	54.4	123.5	65.2	15	14.1	1.2	11	23.3	19.8	12.8	136.5	46.8	523.6
2009-2010	46.6	96.8	52.4	24.8	37.5	2.4	9.1	20.8	10.9	40.3	44.3	56.7	442.6

**Table 3:** Yields of winter wheat under different straw mulch treatments.

**Treatments**	**Grain yield (kg ha^-1^)**		
	**2007-2008**	**2008-2009**	**2009-2010**
SM1	—	4667.7 ± 371.5a	3441.7 ± 118.0a
SM2	—	4434.4 ± 73.6ab	3558.3 ± 225.3a
SM3	—	3975.5 ± 134bc	3375.0 ± 114.1ab
SM4	5674.9 ± 114.8^b^c^a^	3665.8 ± 91.7c	3159.0 ± 40.5b
SM5	5632.5 ± 99.7c	3668.6 ± 205.3c	2900.7 ± 174.8c
SM6	6391.1 ± 191.9a	3682.1 ± 355c	2792.0 ± 128.4c
CK	6033 ± 140.6b	2817.4 ± 277.1d	2683.7 ± 118.5c

Note: CK, no straw mulch; SM1, straw mulch at a high rate of 9000 kg ha^−1^ in whole-period of winter wheat; SM2, straw mulch at a middle rate of 6000 kg ha^−1^ in whole-period of winter wheat; SM3, straw mulch at a low rate of 3000 kg ha^−1^ in whole-period of winter wheat; SM4, straw mulch at a high rate of 9000 kg ha^−1^ in growth-period of winter wheat; SM5, straw mulch at a middle rate of 6000 kg ha^−1^ in growth -period of winter wheat; SM6, straw mulch at a low rate of 3000 kg ha^−1^ in growth -period of winter wheat.

^a^Different lower case letters in the same line indicate significant differences at *P *< 0.05.

^b^Mean ± standard deviation.

**Table 4:** Water consumption, and water-use efficiency (WUE) of winter wheat under different straw mulch treatments.

**Treatments**	**Water consumption (mm)**			**WUE (kg mm^–1^ ha^–1^)**		
	**2007-2008**	**2008-2009**	**2009-2010**	**2007-2008**	**2008-2009**	**2009-2010**
SM1	—	403.0 ± 13.5a	355.9 ± 8.6a	—	11.6 ± 0.2a	9.7 ± 0.1b
SM2	—	390.9 ± 11.8ab	342.1 ± 14.5ab	—	11.3 ± 0.2ab	10.4 ± 0.5a
SM3	—	373.9 ± 10.1bc	347.7 ± 9.2ab	—	10.6 ± 0.6b	9.7 ± 0.2b
SM4	337.9 ± 9.4^b^b^a^	351.3 ± 11.4cd	326.7 ± 12.9b	16.8 ± 0.6ab	10.4 ± 0.9b	9.7 ± 0.4b
SM5	351.2 ± 2.1ab	323.9 ± 13.5d	301.8 ± 9.5c	16 ± 0.6b	11.3 ± 0.3ab	9.6 ± 0.4b
SM6	363.9 ± 8.2a	334.5 ± 14.5d	299.8 ± 15.6c	17.6 ± 0.6a	11 ± 0.4ab	9.3 ± 0.4bc
CK	359.7 ± 9.3a	368.8 ± 24.8bc	301.2 ± 8.0c	16.8 ± 0.3ab	7.6 ± 0.5c	8.9 ± 0.2c

Note: CK, no straw mulch; SM1, straw mulch at a high rate of 9000 kg ha^−1^ in whole-period of winter wheat; SM2, straw mulch at a middle rate of 6000 kg ha^−1^ in whole-period of winter wheat; SM3, straw mulch at a low rate of 3000 kg ha^−1^ in whole-period of winter wheat; SM4, straw mulch at a high rate of 9000 kg ha^−1^ in growth-period of winter wheat; SM5, straw mulch at a middle rate of 6000 kg ha^−1^ in growth -period of winter wheat; SM6, straw mulch at a low rate of 3000 kg ha^−1^ in growth -period of winter wheat.

^a^Different lower case letters in the same line indicate significant differences at *P* < 0.05.

^b^Mean ± standard deviation.

**Table 5:** Basic soil nutrients of the tilth soil (0–60 cm depth) in the experimental site (2007).

**Soil layer (cm)**	**Organic matter**	**Alkali-hydrolyzable nitrogen**	**Total nitrogen**	**Available phosphorus**	**Exchangeable potassium**
	**(g kg^-1^)**	**(mg kg^-1^)**	**(g kg^-1^)**	**(mg kg^-1^)**	**(mg kg^-1^)**
0-20	14.04	53.08	0.69	18.45	141.52
20-40	10.93	34.16	0.55	7.85	100.85
40-60	7.93	26.78	0.44	3.58	83.54
